# Development of a chemically defined cultivation and transfection medium for HEK cell lines

**DOI:** 10.1186/1753-6561-7-S6-P27

**Published:** 2013-12-04

**Authors:** Sebastian Püngel, T Tim Welsink, Penélope Villegas Soto, Wolfgang Weglöhner, Tim F Beckmann, Ina Eickmeier, Stefan Northoff, Christoph Heinrich

**Affiliations:** 1 InVivo BioTech Services GmbH, 16761 Hennigsdorf, Germany; 2 TeutoCell AG, 33615 Bielefeld, Germany

## Background

In the process of generating a production cell, introduction of the gene of interest into the host cell can be performed by various physical, chemical or biological methods. Because of the greater scalability compared to physical methods and no safety concerns or restrictions that are associated with the use of viral systems, a transfection using chemical methods is of great interest. However, up to now up-scaling is limited by the challenge to transfect cells in conditioned media with the widely used reagent polyethylenimine (PEI). Considering the upscaling to gram yields, a culture medium that allows both, transfection and production is required. In this work, the current status in the development of such media supporting cell growth, transfection and protein production in HEK cells is presented. By this, processes will no longer be limited by media exchange prior transient transfection.

## Materials and methods

Transfection was performed according to standard protocols described in the literature. Briefly, 5 × 10^6 ^cells/mL were transfected with 2 pg DNA/cell and 25 kDa PEI in 4 mL transfection volume. Transfection efficiency was determined 24 hours post transfection by counting green fluorescent positive cells using a FACSCalibur (BD Biosciences). All cultivations were carried out using shake flasks with standard conditions well known in the art. Automated viable cell counting was performed by a Cedex (Innovatis). Furthermore, the quantities of components like glucose, lactate, amino acids, salts and vitamins in the supernatant were measured. Based on this information, single ingredients or groups of components from the basal formulation were screened for their influence on transfection efficiency. To evaluate the effect of cellular proteins in conditioned medium, they were separated by chromatography and analyzed via MALDI-TOF/TOF mass spectrometry (MS) (ultrafleXtreme, Bruker). SEC was performed using the high resolution gel filtration medium Superdex™ 200 16/60 with the ÄKTAprime system (GE Healthcare).

## Results

**Figure 1:**
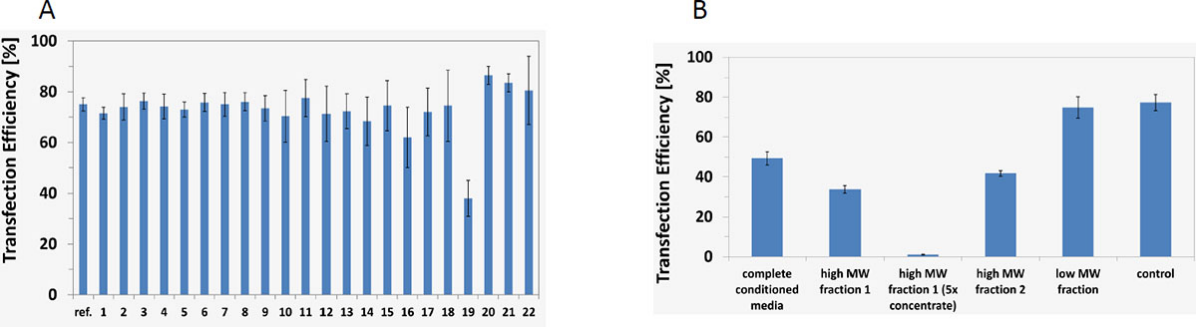
**A: Screening of media components and different concentrations thereof with regard to transfection efficiency**. **B: **Transfection efficiency in conditioned media as well as in fractions from SEC.

Batch growth for an exemplary HEK host cell line in the latest basic growth medium formulation reached a maximum viable cell density of nearly 1 × 10^7 ^cells/mL. Direct adaption of three different adherent serum-depending host cell lines was also successfully implemented in this medium. The screening of basal medium components exhibited no significant influence on transient transfection efficiency of HEK cells (overall efficiency of 80% +/- 15%), as shown in Figure 1(A). In contrast, depending on the level of conditioning, the presence of proteins in the supernatant of these media reduced transfection efficiency up to 100% (Figure 1B).

Separation and analysis of conditioned medium revealed that especially high molecular weight components have a negative impact on the transfection efficiency. Identification by MALDI-TOF/TOF-MS showed not only proteins of the basal lamina but also histones to be present in the analyzed high molecular weight fractions 1 and 2 (Table 1).

**Table 1:** Proteins identified with at least 2 peptides and a false discovery rate of 0% in up to 5 biological replicates by MALDI-TOF/TOF-MS in the high molecular weight fractions 1 and 2.

**High molecular weight fraction 1**			**High molecular weight fraction 2**		
	
**Group**	**Protein name**	**# Peptides**	**Group**	**Protein name**	**# Peptides**
	
Histones	Histone H2A	3	Histones	Histone H2A	4
	Histone H2B	2		Histone H2B	3
	Histone H4	2		Histone H4	4
				Histone H3	3
Cytoskeleton	Tubulin alpha	2			
	Tubulin beta	2	Cytoskeleton	Tubulin alpha	2
	Actin	3		Tubulin beta	3
				Actin	6
Other	Galectin-3-binding protein	6			
	Heat shock 70 kDa protein 1A/1B	5	Extracellular (matrix)	Fibrillin-2	2
				Fibronectin	5
				Clusterin	3
				Cochlin	2
			
			Other	Galectin-3-binding protein	10
				Heat shock 70 kDa protein 1A/1B	13
				Golgi membrane protein 1	6
				Alpha-enolase	2

## Conclusions

The latest medium formulation supports cell growth and easy adaption to suspension of the three major HEK host cell lines and several producer cell lines originated from those. High transfection efficiencies of up to 80% 24 hours post transfection where reached in a basic medium formulation. In this context, the major challenge for combining a transfection- and growth medium in one formulation is to retain single cell growth, while avoiding commonly used anti-aggregation components, which are known to impair transfection efficiency. Beyond that, in this study basal medium components exhibited no influence on transient transfection, whereas high molecular weight fractions of conditioned media reduced transfection efficiency. Noticeably, these fractions contained histones which might be one factor with negative impact.

